# Stepwise positive end-expiratory pressure titration modulates respiratory mechanics and mechanical power in mechanically ventilated adults

**DOI:** 10.62675/2965-2774.20250252

**Published:** 2025-11-01

**Authors:** Adrián Gallardo, Melina Alcaráz, Armando Díaz-Cabrera, Ricardo Arriagada, Patricia Rieken Macedo Rocco, Denise Battaglini

**Affiliations:** 1 Sanatorio Clínica Modelo de Morón Departamento de Kinesiología y Cuidados Respiratorios Buenos Aires Argentina Departamento de Kinesiología y Cuidados Respiratorios, Sanatorio Clínica Modelo de Morón - Buenos Aires, Argentina.; 2 Hospital San Juan de Dios Santiago Chile Hospital San Juan de Dios - Santiago, Chile.; 3 Unidad de Paciente Crítico Hospital Las Higueras de Talcahuano Talcahuano Chile Unidad de Paciente Crítico, Hospital Las Higueras de Talcahuano - Talcahuano, Chile.; 4 Universidade Federal do Rio de Janeiro Laboratory of Pulmonary Investigation Rio de Janeiro RJ Brazil Laboratory of Pulmonary Investigation, Universidade Federal do Rio de Janeiro - Rio de Janeiro (RJ), Brazil.; 5 University of Genova Department of Surgical Sciences and Integrated Diagnostics Genova Italy Department of Surgical Sciences and Integrated Diagnostics, University of Genova - Genova, Italy.

**Keywords:** Mechanical power, Respiration, artificial, Acute respiratory distress syndrome, Critical illness, Lung disease, Positive-pressure respiration, Respiratory mechanics, Ventilator-induced lung injury

## Abstract

**Objective:**

To evaluate the impact of an ascending positive end-expiratory pressure titration strategy on respiratory mechanics and mechanical power in patients without lung injury.

**Methods:**

An incremental positive end-expiratory pressure titration was performed in 4cmH_2_O steps, starting from zero end-expiratory pressure and progressing to 16cmH_2_O. Differences (Δ) in respiratory system static compliance, plateau pressure, driving pressure, and mechanical power were assessed during lung-protective ventilation. Mechanical power formulas proposed by Gattinoni et al. and Costa et al. were used. Analyses were also performed on the static elastic components, dynamic elastic components, total elastic power, and resistive components.

**Results:**

Increasing positive end-expiratory pressure levels were associated with a progressive rise in mechanical power, plateau pressure, total and static elastic power, and a decline in compliance. Mechanical power showed strong positive correlations with: ΔPplat (p < 0.001); Δelastic dynamic power (p < 0.001); Δdriving pressure (p < 0.001); and Δtotal elastic power (p < 0.001). Δmechanical power correlated strongly with Δresistive power (p < 0.001), but not with other mechanical power components or mechanics.

**Conclusion:**

Progressive positive end-expiratory pressure increase in patients without lung disease significantly raises total mechanical power and its elastic components, particularly static elastic power. These changes may occur silently and without significant alterations in driving pressure or compliance.

## INTRODUCTION

Lung-protective mechanical ventilation (MV) is a cornerstone in the management of critically ill patients.^(1)^ This strategy involves carefully adjusting and monitoring multiple ventilatory variables to optimize gas exchange while minimizing injury to both the lungs^(2)^ and the diaphragm.^(3)^ Mechanical ventilation induces stretching of the lung parenchyma through both static and dynamic components: the static component is primarily influenced by the application of positive end-expiratory pressure (PEEP),^(4)^ whereas the dynamic component results from the cyclic delivery and removal of tidal volume (VT).^(5)^

A significant challenge in clinical practice is distinguishing between alveolar recruitment and overdistension caused by PEEP. While PEEP may reopen collapsed alveoli, it can also distend already aerated regions, potentially exacerbating ventilator-induced lung injury (VILI).^(5)^ Nonetheless, there likely exists a non-injurious lung volume – a physiological balance point between PEEP and functional residual capacity – at which optimal respiratory mechanics and minimal mechanical power (MP) are achieved. Mechanical power, the energy delivered to the respiratory system per unit time, encompasses static and dynamic elastic components.^(5)^

Lung overdistension may also impose downward force on the diaphragm, altering its geometry and placing it at a mechanical disadvantage for force generation. This can impair diaphragmatic contractility and contribute to longitudinal atrophy during prolonged MV.^(6)^ Elevated intrathoracic pressures from high PEEP levels can increase pulmonary vascular resistance, reduce venous return, and compromise hemodynamics. Thus, the net effect of PEEP depends on the balance between recruitment and overdistension, which ultimately determines whether its application is protective or injurious.

Despite extensive investigation, the optimal PEEP level remains unclear, particularly in patients with non-injured lungs.^(7,8)^

To address this knowledge gap, we conducted a physiological study of 16 deeply sedated adult patients (Richmond Agitation-Sedation Scale [RASS] score of −5) without pre-existing lung disease, within the first 48 hours of MV. We assessed the effects of stepwise PEEP titration (0, 4, 8, 12, and 16cmH_2_O) on respiratory mechanics and mechanical power (dynamic and static components). Our objective was to evaluate the impact of an ascending PEEP titration strategy on respiratory mechanics and MP in patients without lung injury. As a secondary objective, we evaluated the correlations between MP components and key respiratory parameters (e.g., respiratory system compliance [Crs], driving pressure [DP], and airway resistance) to explore potential mechanistic interdependencies.

## METHODS

The study was conducted according to the Declaration of Helsinki and approved by the institutional ethics committee of *Sanatorio Clínica Modelo de Morón*, Argentina (Approval No. 02/2024). Informed consent was obtained from all participants in accordance with local regulations.

### Positive end-expiratory pressure titration protocol

An incremental PEEP titration test was performed in 4cmH_2_O steps, starting from zero end-expiratory pressure (ZEEP, 0cmH_2_O) and progressing to 16cmH_2_O. Each PEEP level was maintained for 10 minutes to allow the respiratory system to reach a steady state before measurements were taken.

Respiratory system static compliance, plateau pressure (Pplat), DP (defined as Pplat – total PEEP), and MP were assessed at the end of each step. Mechanical ventilation was delivered in volume control ventilation mode, with VT set between 6 and 8mL/kg of predicted ideal body weight. The respiratory rate (RR) was adjusted to maintain arterial carbon dioxide partial pressure (PaCO_2_) between 35 and 45mmHg. The fraction of inspired oxygen (FiO_2_) was set to the minimum value required to maintain SpO_2_ ≥ 94%. All patients were assessed in the supine position with a 45° head-of-bed elevation.

At each PEEP level, Pplat, peak inspiratory pressure (PIP), and DP were measured using a 3-second end-inspiratory pause. Respiratory system compliance was calculated as VT/DP.

### Mechanical power calculations

Mechanical power and its components were calculated using the following formulas:

- Mechanical power from Costa et al.^(9)^ = MP_COSTA_ = 4 × DP + RR
- Total mechanical power from Gattinoni et al.^(10)^ = MP_GATTINONI_ = 0.098 × RR × VT × (PIP − 0.5 × DP)
- Total elastic power = 0.098 × VT × RR × 0.5 × (Pplat + PEEP)
- Elastic dynamic power = 0.098 × VT × RR × 0.5 × (Pplat − PEEP)
- Elastic static power = 0.098 × VT × RR × PEEP
- Resistive power = 0.098 × VT × RR × (PIP − Pplat)

### Statistical analysis

Qualitative variables are reported as absolute frequencies and percentages (n; %), while quantitative variables are presented as mean ± standard deviation (SD). Normality was assessed using the Shapiro-Wilk test (for n < 50) or the Kolmogorov–Smirnov test (for n ≥ 50).

Comparisons across PEEP levels (0, 4, 8, 12, and 16cmH_2_O) were performed using repeated-measures analysis of variance (ANOVA), after confirming sphericity (Mauchly's test) and normality of residuals (Shapiro–Wilk test *per PEEP level*). When sphericity was violated (p < 0.05), Greenhouse–Geisser correction was applied. Bonferroni-adjusted *post hoc* tests were used for pairwise comparisons (e.g., PEEP 4cmH_2_O *versus* PEEP 0cmH_2_O). Paired t-tests were used to compare MP, elastic dynamic power, and elastic static power between adjacent PEEP levels.

To evaluate associations between MP components and respiratory mechanics parameters (e.g., Crs, DP, resistance), Spearman's rank correlation coefficient (ρ) was computed. Correlations were based on the differences (Δ) between consecutive PEEP levels (0→4→8→12→16cmH_2_O), grouped by variable (n = 63). An additional correlation matrix was constructed comparing changes from baseline (PEEP = 0cmH_2_O) for each variable (0 *versus* all: 4→8→12→16cmH_2_O).

All analyses were performed using GraphPad Prism 9, with a two-tailed significance level set at p < 0.05 and 95% confidence intervals (95%CI).

## RESULTS

### Baseline characteristics

Table 1 summarizes baseline patient characteristics. The mean age was 57 years (SD = 16), and the majority were male (81.25%). All patients were ventilated using a lung-protective strategy, with a mean VT of 7.3 ± 0.6mL/kg of predicted body weight.

**Table 1:** Baseline characteristics

Variable		
Age (years)		57.1 ± 16.3
Male sex		13 (81.3)
Height (cm)		172.6 ± 8.5
Weight (kg)		67.6 ± 8.6
APACHE II		21.6 ± 4.4
Reasons for mechanical ventilation		
	Consciousness deterioration	5 (31.25)
	Cardiovascular surgery	3 (18.75)
	Abdominal surgery	4 (16.0)
	Head trauma	2 (8.0)
	Cardiorespiratory arrest	2 (8.0)
Days on mechanical ventilation		1.2 ± 0.4
Orotracheal tube diameter (mm)		8.1 ± 0.2
Ventilatory mode (VC-CMV), n (%)		16 (100)
Tidal volume (mL)		492.5 ± 50.4
Respiratory rate (breaths/minute)		18.4 ± 2.1
FiO_2_		36.3 ± 7.7
Peak flow (L/minute)		60 ± 2.3
Tidal volume/ideal weight (mL/kg)		7.3 ± 0.6

APACHE II - Acute Physiology and Chronic Health Evaluation II; VCV: volume-controlled ventilation; FiO_2_ - inspiratory oxygen fraction. Data are presented as mean ± standard deviation or absolute frequency (%).

### Energy transmission during mechanical ventilation

As shown in table 2, energy-related parameters changed significantly with increasing PEEP levels.

**Table 2:** Variables related to energy transmission during mechanical ventilation, driving pressure, and working pressure and its components at different levels of positive end-expiratory pressure

Variables	PEEP 0	PEEP 4	PEEP 8	PEEP 12	PEEP 16	ANOVA p value
MP (J.min^−1^) Gattinoni et al.^(10)^	12.3 ± 3.6	14.7 ± 3.9*	17.8 ± 3.9*	21.3 ± 4.2*	25.7 ± 5.0*	< 0.001
MP (J.min^−1^) Costa et al.^(9)^	61.6 ± 7.2	63.4 ± 9.0	65.4 ± 9.6	68.6 ± 12.4	70 ± 9.6	< 0.001
Total elastic power (J.min^−1^)	4.8 ± 0.8	8.5 ± 1.1*	12.2 ± 1.3*	16.2 ± 2*	19.8 ± 2.2*	< 0.001
Dynamic elastic power (J.min^−1^)	4.8 ± 0.8	5.0 ± 0.9	5.2 ± 1	5.6 ± 1.2	5.7 ± 1.1	< 0.001
Static elastic power (J.min^−1^)		3.5 ± 0.4	7.1 ± 0.7*	10.6 ± 1.1*	14.1 ± 1.5*	< 0.001
Resistive power (J.min^−1^)	7.5 ± 3.9	6.1 ± 4.1*	5.5 ± 4.0	4.9 ± 4.2	5.8 ± 4.6	< 0.001
Peak pressure (cmH_2_O)	19.4 ± 3.7	22.3 ± 3.7*	26 ± 3.4*	30.4 ± 3.7*	35.7 ± 4.7*	< 0.001
Crs (mL.cmH_2_O^−1^)	51.1 ± 12.1	53.8 ± 13.4	47.2 ± 12.5*	45.5 ± 9.5	43.7 ± 9.9	0.008
Driving pressure (cmH_2_O)	10.8 ± 1.8	11.3 ± 2.2	11.8 ± 2.4	12.6 ± 3.0	12.9 ± 2.4	< 0.001
Plateau pressure (cmH_2_O)	10.8 ± 1.8	15.4 ± 2.0*	19.8 ± 2.3*	24.8 ± 2.9*	29.1 ± 2.1*	< 0.001

PEEP - positive end-expiratory pressure; MP - mechanical power; Crs - respiratory system static compliance. Variables related to energy transmission during mechanical ventilation across positive end-expiratory pressure levels. One-way ANOVA with Bonferroni post hoc test was used;

* Significant difference *versus* preceding positive end-expiratory pressure level (p < 0.05). Data are presented as mean ± standard deviation.

MP_GATTINONI_ increased progressively from 12.3 ± 3.6 J·min^-1^ at PEEP 0 to 25.7 ± 5.0 J·min^-1^ at PEEP 16 (p < 0.001). Relative increases were consistent across steps: +19.5% (PEEP 0→4), +21.1% (4→8), +19.7% (8→12), and +20.7% (12→16). In contrast, MP_COSTA_ increased from 61.6 ± 7.2 to 70.0 ± 9.6 J·min^-1^ (p < 0.001), with smaller and statistically nonsignificant increments across individual PEEP steps: +2.9% (0→4), +3.2% (4→8), +4.9% (8→12), and +2.0% (12→16).

### Elastic power components showed divergent trends

Total elastic power increased exponentially, from 4.8 ± 0.8 J·min^-1^ (PEEP 0) to 19.8 ± 2.2 J·min^-1^ (PEEP 16) (p < 0.001), with significant increases at each step: +73.5%, +43.5%, +32.8%, and +22.2%, respectively. Elastic dynamic power showed a modest increase from 4.8 ± 0.8 to 5.7 ± 1.1 J·min^-1^ (p < 0.001), with no significant pairwise differences between steps. Elastic static power, which could not be quantified at PEEP 0, reached 14.1 ± 1.5 J·min^-1^ at PEEP 16, with marked increases across all subsequent steps (p < 0.001). Resistive power initially decreased by 18.7% from PEEP 0 to 4 (p < 0.05), followed by further reductions from PEEP 4→8 (−9.8%) and 8→12 (−10.9%), and a nonsignificant rebound from PEEP 12→16 (+18.4%).

### Pressures and respiratory mechanics

All respiratory pressures rose progressively with PEEP (Table 2): PIP increased from 19.4 ± 3.7 to 35.7 ± 4.7cmH_2_O (p < 0.001), with consistent increases at each step: +14.9%, +16.6%, +16.9%, and +17.4%. Plateau pressure doubled from 10.8 ± 1.8 to 29.1 ± 2.1cmH_2_O (p < 0.001), with progressively smaller percentage increases: +42.6%, +28.6%, +25.3%, and +17.3%. Respiratory system compliance peaked at PEEP 4 (53.8 ± 13.4mL·cmH_2_O^-1^), representing a 5.3% increase from baseline. It subsequently declined to 43.7 ± 9.9mL·cmH_2_O^-1^ at PEEP 16 (p = 0.008), with significant reduction from PEEP 4→8 (−12.3%) and smaller nonsignificant decreases thereafter (−3.6% and −4.0%).

**Figure 1:**
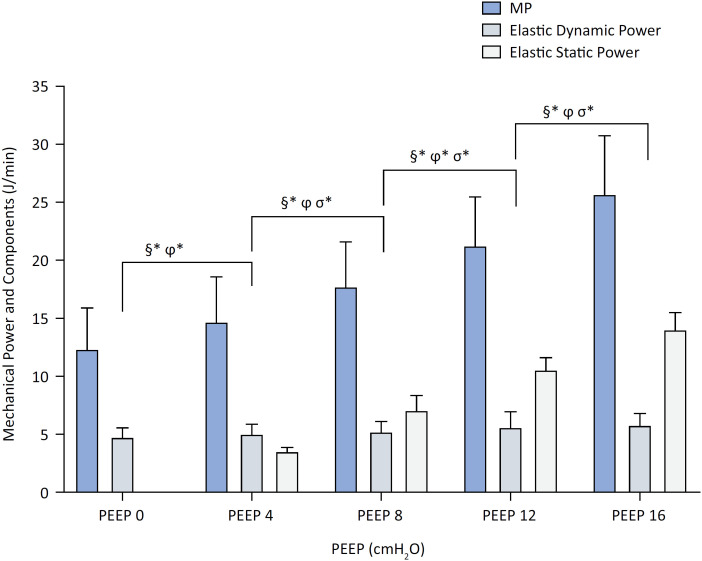
Shows mechanical power, elastic dynamic power and elastic static power across incremental positive end-expiratory pressure levels (0, 4, 8, 12, 16cmH_2_O).

Figure 1 illustrates the evolution of MP and its components across PEEP levels. Mechanical power (§) increased steadily with significant differences between all steps (p < 0.001), while Elastic static power (σ) increased markedly (p < 0.001). Elastic dynamic power (ϕ) showed significant changes only from PEEP 0→4 (p = 0.04) and 8→12 (p = 0.03).

MP - mechanical power; PEEP - positive end-expiratory pressure.

* Statistically significant differences (p < 0.05) between consecutive positive end-expiratory pressure levels, as determined by paired t-tests; § mechanical power; ϕ elastic dynamic power; σ elastic static power.

### Correlations between energy transmission and lung mechanics

**Figure 2:**
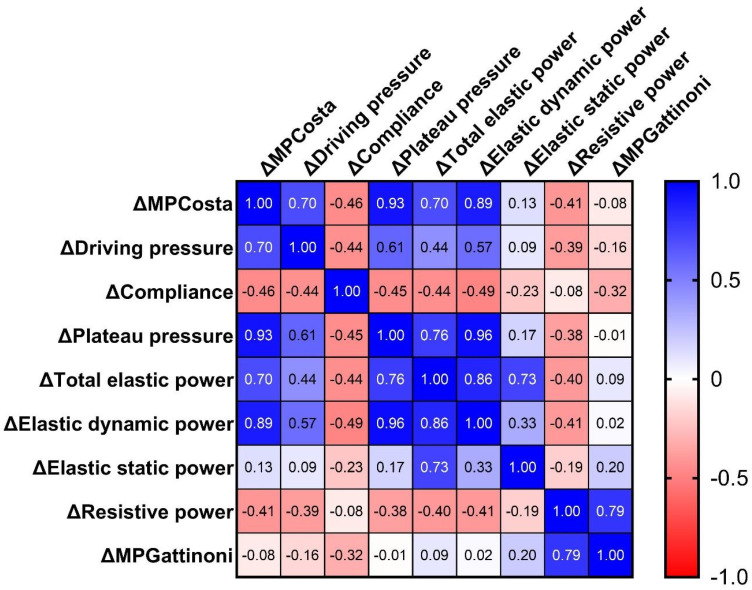
Spearman's correlation matrix of Δ-mechanical power components and Δ-lung mechanics between consecutive positive end-expiratory pressure levels (0→4→8→12→16cmH_2_O).

Figure 2 shows the Spearman correlation matrix for differences (Δ) in MP and lung mechanics across PEEP levels (0→4→8→12→16cmH_2_O).

MP - mechanical power.

The matrix displays pairwise Spearman's rank correlation coefficients (ρ) for changes (Δ) between successive positive end-expiratory pressure steps in:

– Mechanical power components: Δtotal elastic power, Δelastic dynamic power, Δelastic static power, Δresistive power, ΔMP_COSTA_^(9)^ and ΔMP_GATTINONI_.^(10)^

– Lung mechanics: Δdriving pressure, Δrespiratory system compliance, Δplateau pressure.

MP_COSTA_ showed strong positive correlations with: ΔPplat (ρ = 0.93; 95%CI: 0.88 - 0.96; p < 0.001); ΔElastic dynamic power (ρ = 0.90; 95%CI: 0.82 - 0.93; p < 0.001); ΔDriving pressure (ρ = 0.70; 95%CI: 0.54 - 0.81; p < 0.001); and ΔTotal elastic power (ρ = 0.70; 95%CI: 0.54 - 0.81; p < 0.001). It also showed a moderate inverse correlation with ΔCrs (ρ = −0.46; 95%CI: −0.64 to −0.23; p < 0.001), suggesting that increasing PEEP raises energy expenditure at the cost of reduced Crs.

ΔMP_GATTINONI_ correlated strongly with ΔResistive power (ρ = 0.79; 95%CI: 0.68 - 0.87; p < 0.001), but not with other MP components or mechanics. A weak negative correlation was found with ΔCrs (ρ = −0.32; 95%CI: −0.53 to −0.07; p = 0.01).

ΔCrs showed significant negative correlations with ΔMP_COSTA_, ΔDP (ρ = −0.44), and ΔPplat (ρ = −0.45), confirming that Crs declines as pressures and energy increase.

**Figure 3:**
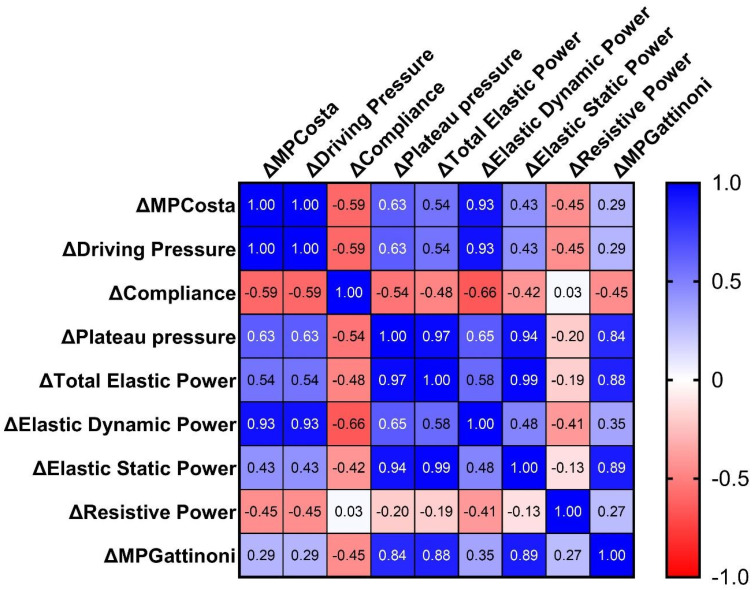
Spearman's correlation matrix of Δ-mechanical power components and Δ-lung mechanics parameters (reference: positive end-expiratory pressure 0).

Figure 3 shows a secondary matrix using baseline (PEEP 0) as reference (0 *versus* all: 4→8→12→16cmH_2_O): ΔMP_COSTA_ showed perfect correlation with ΔDP (ρ = 1.0; 95%CI: 1 - 1; p < 0.001) and strong associations with ΔElastic dynamic pressure (ρ = 0.94), ΔPplat (ρ = 0.63), and inverse correlation with ΔCrs (ρ = −0.59). ΔMP_GATTINONI_ correlated strongly with ΔTotal elastic power (ρ = 0.88), ΔElastic static power (ρ = 0.89), and ΔPplat (ρ = 0.84), reinforcing that this model reflects changes in both resistive and elastic energy, especially static components.

MP - mechanical power.

The matrix displays pairwise Spearman's rank correlation coefficients (ρ) for changes (Δ) from positive end-expiratory pressure 0 to higher positive end-expiratory pressure levels (4→8→12→16cmH_2_O) in:

– Mechanical power components: Δtotal elastic power, Δelastic dynamic power, Δelastic static power, δresistive power, ΔMP_COSTA_^(9)^ and ΔMP_GATTINONI_.^(10)^

– Lung mechanics parameters: Δdriving pressure, Δrespiratory system compliance, Δplateau pressure.

## DISCUSSION

Our findings demonstrate that increasing PEEP levels in mechanically ventilated patients without lung disease is associated with a progressive rise in MP, Pplat, total and static elastic power, and a concurrent decline in Crs. Notably, even in this population without pre-existing pulmonary pathology, PEEP titration in 4cmH_2_O increments resulted in MP values exceeding thresholds previously proposed for lung protection.^(11,12)^ These modifications in respiratory mechanics variables may respond to the characteristics of healthy lungs, where recruitment potential is usually low. This would explain the drop in compliance (ΔVT/DP) when the variable located in the denominator of the formula that defines it increases. At the same time, the total mechanical power would move in the opposite direction, since its static component increased, and its dynamic component remained stable.

These results are partially consistent with previous findings^(13)^ in pediatric population – 45% of whom had moderate acute respiratory distress syndrome (ARDS) – and found that PEEP levels of 10 - 12cmH_2_O were associated with substantial increases in MP (60%) and DP (33%). In contrast, our adult patients exhibited a gradual and significant increase in Pplat across all PEEP steps, peaking at PEEP 16cmH_2_O. However, DP increased only modestly and not significantly, while Crs steadily declined from PEEP 4cmH_2_O onward. Importantly, from PEEP 8cmH_2_O, the static component of elastic power began to predominate, indicating a shift in the energy load profile toward sustained alveolar distension. Notably, this change (and subsequent ones) did not impact the hemodynamic status.

While Ferraz et al.,^(13)^ reported a moderate positive correlation between MP and DP (ρ = 0.59), our study observed a strong correlation only with Costa et al.'s^(9)^ MP (ρ = 0.7), not with MP Gattinoni et al.'s formula^(10)^ (ρ = −0.16). This discrepancy likely reflects differences in the underlying assumptions of each formula. Gattinoni et al.'s MP^(10)^ incorporates flow and RR components, which may be less influential in patients with preserved lung mechanics. In contrast, Costa et al.'s model^(9)^ – focusing on elastic load and distending pressure – may more accurately reflect changes in Crs even in the absence of overt lung injury. Furthermore, differences in study populations, body weight normalization, and pathophysiological context may account for these divergent findings. These considerations underscore the importance of selecting an MP formula tailored to the patient's physiological profile.

Importantly, our results reinforce the relevance of deconstructing MP into its elastic and resistive components to better understand the mechanical burden imposed on the lungs. In both MP models, total mechanical power increased with higher PEEP levels, but only Gattinoni et al.'s model^(10)^ showed significant differences between consecutive PEEP steps. This likely stems from its greater sensitivity to flow-related changes. However, the elastic component – especially the static portion – accounted for the most pronounced changes. Elastic dynamic power remained relatively stable, suggesting that the primary contributor to rising energy load was sustained static distension rather than tidal recruitment.

This pattern aligns with findings from the MP day study,^(14)^ in which a *post hoc* analysis demonstrated that elastic static power correlated more strongly with ARDS severity than other MP components.^(15)^ In their analysis, elastic static power values exceeding 4.8 J/min were associated with higher ARDS severity. In our patients without lung injury, elastic static power exceeded 4.8 J/min as early as PEEP 4cmH_2_O and reached 14.1 J/min at PEEP 16cmH_2_O. This raises important concerns regarding potential alveolar overdistension and subclinical VILI, even in healthy lungs.

Interestingly, we observed that from PEEP 8cmH_2_O, the static elastic component surpassed the dynamic component – whereas in Ferraz et al.'s pediatric cohort, the dynamic component remained dominant across all PEEP levels. These differences may be attributed to the study population (adult *versus* pediatric), absence of lung disease in our cohort, lack of body weight normalization, and anatomical and physiological differences between adult and pediatric pulmonary systems.^(13)^

The implications are twofold. First, our data confirm that MP is not solely a function of tidal volume or RR but is profoundly affected by PEEP. Second, static elastic power – often underappreciated – may represent a crucial determinant of alveolar strain. These insights are relevant for patients with lung injury and those with preserved pulmonary mechanics, where elevated PEEP may impose unintended mechanical stress.

Moreover, despite the absence of ARDS, our patients exhibited MP values and PEEP-related changes similar to those reported in moderate or severe ARDS cohorts.^(15)^ For instance, in Fajardo-Campoverdi et al.,^(15)^ Pplat ranged from 17.4 to 20.5cmH_2_O depending on ARDS severity. In our cohort, several patients exceeded 30cmH_2_O, despite a stable RR and controlled settings. This highlights the risk of energy overload through seemingly moderate ventilatory adjustments, even in non-injured lungs.

Notably, PEEP 8cmH_2_O marked the transition point where MP surpassed 17 J/min – considered the upper safety limit in healthy lungs^(11)^ – and where the static elastic component began to dominate. This suggests a physiologically meaningful threshold, beyond which the risk of lung injury may rise disproportionately. The observed strong correlations between ΔPplat, ΔMP, and Costa's^(9)^ MP reinforce the clinical utility of this model in capturing elastic load increases due to rising PEEP.

### Strengths and limitations

This study offers novel insights into the differential effects of increasing PEEP on mechanical power and its components in patients without lung disease – a population often underrepresented in MV research. Using both Gattinoni et al.'s^(10)^ and Costa et al.'s^(9)^ formulas allowed for a comprehensive comparison and a nuanced understanding of how elastic and resistive loads evolve with PEEP.

However, several limitations must be acknowledged. The sample size was small and lacked power for subgroup analysis, limiting generalizability. The short duration (10 minutes) of each PEEP step may not have allowed complete equilibration. Arterial blood gases and direct measures of alveolar recruitment (e.g., via electrical impedance tomography) were not included. Moreover, we did not normalize MP to body weight, which may affect comparisons with pediatric and ARDS populations. Additionally, hemodynamic data were not collected during the study, despite no patient exhibiting impairment or instability. Finally, the study's relevance is limited to patients with preserved pulmonary function.

### Clinical implications

These findings carry important clinical implications. In patients without apparent lung pathology, inappropriate PEEP titration may result in excessive static energy transfer and potential overdistension. While informative, standard bedside parameters such as DP and Pplat may not fully reflect the underlying mechanical burden. Integrating mechanical power assessment – particularly the static elastic component – could improve ventilator settings and reduce the risk of occult VILI. Formulas like Costa et al.'s^(9)^ may offer more physiologically relevant insights in patients with relatively normal respiratory system resistance.

Our results also support the practice of individualized PEEP titration based on gas exchange or compliance and energy load parameters.^(16)^ A strategy favoring lower PEEP (e.g., 4cmH_2_O) provided the lowest MP, highest Crs, and minimized the static load, suggesting a potential lung-protective approach even without lung injury.

## CONCLUSION

Progressive positive end-expiratory pressure elevation in patients without lung disease significantly increases total mechanical power and its elastic components – particularly static elastic power – often exceeding thresholds considered protective. These changes occur even without significant alterations in driving pressure or respiratory system static compliance. The predominance of the static component at higher positive end-expiratory pressure levels suggests that overdistension may develop silently in such patients. Routine monitoring of mechanical power, with attention to its elastic partitioning and the use of physiologically appropriate models, may enhance lung-protective ventilation strategies and help prevent ventilator-induced lung injury, even in seemingly healthy lungs.

## AVAILABILITY OF DATA AND MATERIALS

After publication the data will be available on demand to authors.
